# Complete genome sequences of cluster G1 and cluster K4 *Mycobacterium smegmatis* phages omnicritical and Barkley26

**DOI:** 10.1128/MRA.00274-23

**Published:** 2023-09-06

**Authors:** Ojas Sali, Dave M. Patel, Michaelangelo C. Ortega, Sydnee Han, Hanford Gerille Esperanza Gonzales, Omar Sulaiman Haneefzai, Carlos Herrera, Jireh Kim, Brooke Knoles, Abigail Lopez, Justin Aaron Richardson, David Andres Roach, Maxene Vergonia-Fehlman, Jessica Vincent, Sang Ho Yim, Leo I. C. Cutter, Christy Strong, Philippos K. Tsourkas, Kurt Regner

**Affiliations:** 1 School of Life Sciences, University of Nevada, Las Vegas, Nevada, USA; 2 School of Medicine and Public Health, University of Wisconsin, Madison, Wisconsin, USA; Department of Biology, Queens College, New York, New York, USA

**Keywords:** *Mycobacterium*, DNA master, phage commander, actinobacteriophages

## Abstract

We present the complete genome sequence of two Actinobacteriophages, OmniCritical and Barkley26, isolated in Clark County, NV. Over two semesters, The University of Nevada, Las Vegas (UNLV) students isolated and purified phages and manually annotated the genomes. The courses follow the HHMI Science Education Alliance Phage Hunters Advancing Genomics and Evolutionary Sciences (SEA-PHAGES) curricula.

## ANNOUNCEMENT

OmniCritical was isolated from a compost tumbler at a private residence (36.131376 N, 115.240078 W) and Barkley26 from the soil of a polka dot plant (*Hypoestes phyllostachya*) grown at UNLV (36.10983 N, 115.14321 W). Phage isolation and purification followed the HHMI Phage Discovery Guide protocols with *Mycobacterium smegmatis* mc^2^ 155 as the host (1). Phages were considered pure after three successive plaque assays produced plaques of uniform diameter and shape. High titer (10^10^–10^11^) lysates were generated for DNA isolation and transmission electron microscopy (TEM). Genomic DNA was isolated using a commercial kit (Norgen Biotek), and both DNA samples were sequenced with the Illumina MiSeq System. The total reads for OmniCritical (SRX14483223) and Barkley26 (SRX14443484) were 777,403 and 737,822, respectively. Newbler (v. 2.9, 454 Life Science) was used for *de novo* assembly of the raw reads, generating a single contig as described in reference (2). Sequence completeness, accuracy, and phage genomic termini were assessed using Consed (v. 29) (3). In preparation for TEM, copper 300 mesh grids (Ted Pella, Inc.) were spotted with 10 µL of phage lysate and stained with 1% phosphotungstate (Electron Microscopy Services). TEM was performed at 120 keV on a JEOL JEM-1400 Plus at the Electron Microscopy Core Laboratory, University of Utah. Images were acquired with DigitalMicrograph with a Gatan Orius SC1000 CCD camera (1 s acquisition time).

From FASTA files, putative genes were identified with DNA Master (v5.38.8) and Phage Commander, which summarize query results from Glimmer (v3.02b), Genemark (v2.5), Genemark Hmm (v2.0), GenemarkS (v4.28), GenemarkS2, Genemark Heuristic (v.2.0), Prodigal (v2.6.3), RAST (v2.0), and Metagene (v2.0) (3–13). Putative genes and their start codons were evaluated using the method previously described (14). Putative functions were assigned using BLAST (10^−7^ *E*-value cutoff), CD-Search (0.001 *E*-value threshold), and HHPred (<0.0001 *E*-value); Aragorn was used to identify tRNA genes (15–18). Default parameters were used for all software unless otherwise specified. Phage cluster and subcluster assignments were determined with Phamerator (19) using the protocols in reference (20). OmniCritical and Barkley26 are part of the fifth set of phages discovered at UNLV; since the course was first offered in 2017–2018, a total of 11 *M*. *smegmatis* phages have now been discovered and published (21–24). Interestingly, OmniCritical belongs to the K4 subcluster, to which phage Reptar3000, discovered at UNLV in 2018, also belongs. The GenBank and SRA accession numbers, average coverage depth, cluster assignment, and genome analyses appear in Table 1.

**TABLE 1:** Phage GenBank and SRA accession numbers and genome assembly results

Phage name	GenBank accession no.	SRA accession no.	Average coverage (×)	Cluster and subcluster	Genome length (bp)	GC content (%)	No. of genes
Barkley26	ON645337.1	SRX14443484	2,499×	G G1	41,756	66.6%	62
OmniCritical	ON645342.1	SRX14483223	1,894×	K K4	57,973	68.0%	95

**Fig 1:**
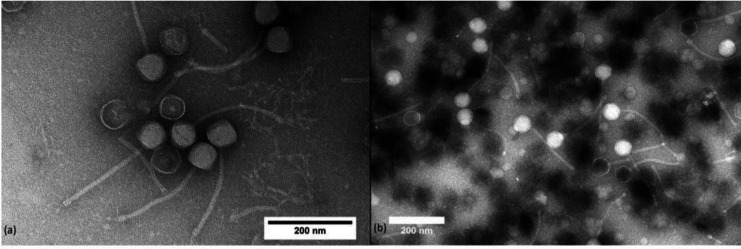
Transmission electron microscopy of *Mycobacterium smegmatis* bacteriophages (a) OmniCritical and (b) Barkley26 was performed at 120 keV on a JEOL JEM-1400 Plus at the Electron Microscopy Core Laboratory at the University of Utah. The images were captured by DigitalMicrograph with a Gatan Orius SC1000 CCD camera (1 s acquisition time) after being negatively stained with 10 µL of 1% phosphotungstate.

OmniCritical and Barkley26 are temperate Siphoviruses that produced 4.0 and 5.0 mm diameter plaques, respectively. The capsid diameter and tail length of OmniCritical are 79.1 and 232.6 nm; for Barkley26, these dimensions are 58.6 and 245.3 nm (Fig. 1). OmniCritical has 95 genes, 47 of which have an assigned protein function, with one being a tRNA-lysine gene. Barkley26 has 62 genes, and 29 have assigned protein functions. Both phages contain a programmed translational frameshift in the tail assembly chaperone protein, which was annotated according to the method described in reference (3). Both phages encode a tyrosine integrase, Cro, and an excisionase, indicating they are temperate. A complete list of genes and functions for both phages is available at The Actinobacteriophage Database (https://phagesdb.org).
